# Crystal structures of the polymer precursors 3-(2,5-dimeth­oxy-3,4,6-tri­methyl­phen­yl)propyl methacrylate and 3-(2,4,5-trimethyl-3,6-dioxo­cyclo­hexa-1,4-dien­yl)propyl methacrylate

**DOI:** 10.1107/S2056989017004959

**Published:** 2017-04-04

**Authors:** Shailesh K. Goswami, Lyall R. Hanton, C. John McAdam, Stephen C. Moratti, Jim Simpson

**Affiliations:** a Department of Chemistry, University of Otago, PO Box 56, Dunedin, New Zealand

**Keywords:** crystal structure, methacrylate, di­meth­oxy­benzene, quinone, hydrogen bonds, C—H⋯π contacts

## Abstract

The closely related title compounds, 3-(2,5-dimeth­oxy-3,4,6-tri­methyl­phen­yl)propyl methacrylate, C_18_H_26_O_4_ (I), and 3-(2,4,5-trimethyl-3,6-dioxo­cyclo­hexa-1,4-dien­yl)propyl methacrylate, C_16_H_20_O_4_ (II), are monomers suitable for the preparation of redox polymers. They consist of a propyl­methacrylate group and three methyl substituents on di­meth­oxy­benzene and quinone cores, respectively. Both crystal structures feature weak C—H⋯O hydrogen bonds and C—H⋯π(ring) contacts between methyl groups and the six-membered rings.

## Abstract 2

The mol­ecular and crystal structures of 3-(2,5-dimeth­oxy-3,4,6-tri­methyl­phen­yl)propyl methacrylate and 3-(2,4,5-trimethyl-3,6-dioxo­cyclo­hexa-1,4-dien­yl)propyl methacrylate, synthesized as precursors to redox-active polymer gel systems, are reported.

## Chemical context   

The title compounds, (I) and (II), were synthesised as part of our continuing inter­est in redox polymers and electrochemical actuators (Dana *et al.*, 2007 ▸; McAdam *et al.*, 2008 ▸; Goswami *et al.*, 2013 ▸, 2015 ▸). Redox-active polymers containing 2,2,6,6-tetra­methyl­piperidin-1-oxyl-4-yl (TEMPO) and ferrocene as pendant groups are well documented (Gracia & Mecerreyes, 2013 ▸; Tamura *et al.*, 2008 ▸; Schattling *et al.*, 2014 ▸). In contrast, polymers with pendant quinone units are less well explored (Hodge & Gautrot, 2009 ▸; Häupler *et al.*, 2014 ▸). Reasons for this include their free-radical-scavenging properties in free radical polymerization (FRP), and the incompatibility of the quinone carbonyl groups in typical living polymerization such as anionic or cationic polymerization. In previous work (Goswami *et al.*, 2013 ▸) we successfully demonstrated that steric hindrance by alkyl groups around a quinone unit prevents radical addition to the ring or the carbonyl oxygen atom, thus enabling FRP synthesis of homo- and co-polymers of quinone-appended methacrylate monomers.

## Structural commentary   

**Figure 1:**
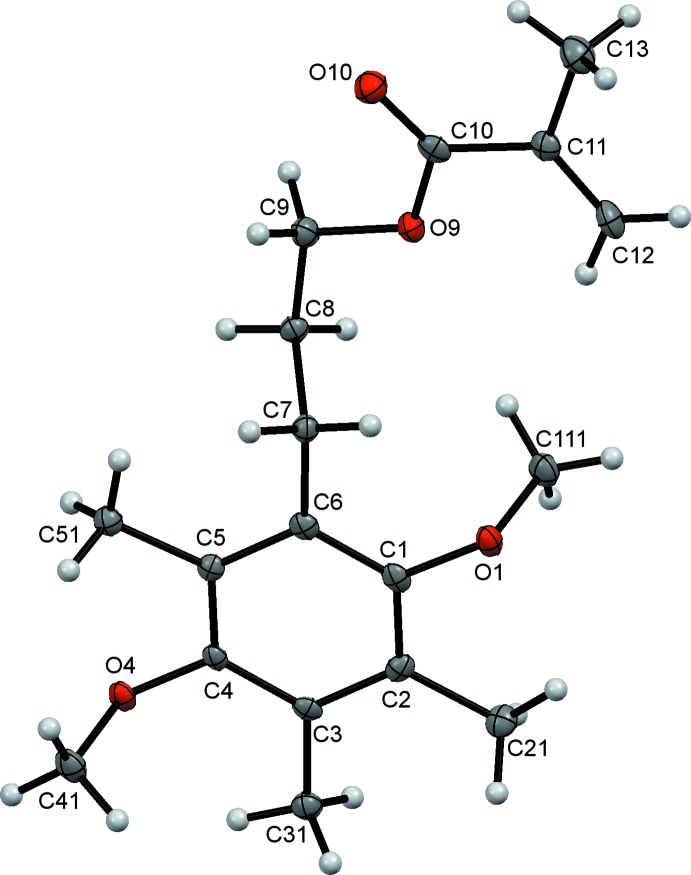
The mol­ecular structure of compound (I), with displacement ellipsoids drawn at the 50% probability level.

**Figure 2:**
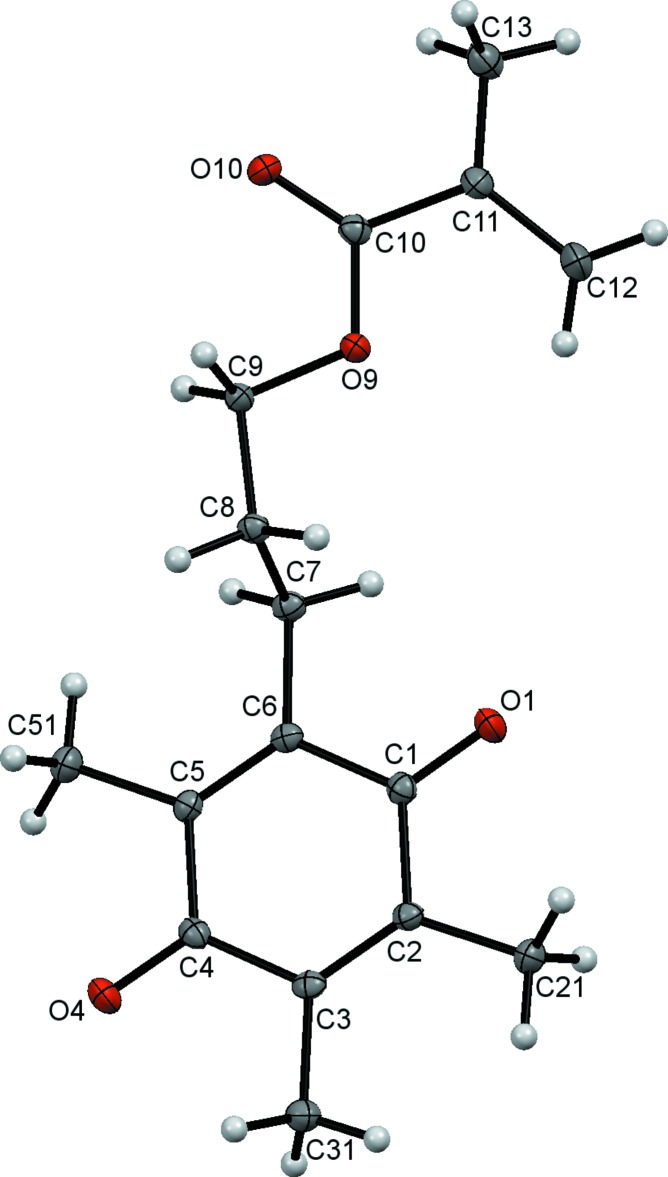
The mol­ecular structure of compound (II), with displacement ellipsoids drawn at the 50% probability level.

Compound (I), a tetra-alkyl­ated *p*-di­meth­oxy­benzene is shown in Fig. 1 ▸. The meth­oxy substituents are in the typical *trans* conformation (Wickramasinhage *et al.*, 2016 ▸; Wiedenfeld *et al.*, 2003 ▸; Wieczorek *et al.*, 1975 ▸) with a C111—O1⋯O4—C41 torsion angle of approximately 179.24°. Three methyl groups and a propyl methacrylate occupy the other four sites on the benzene ring. Compound (II), shown in Fig. 2 ▸, is the quinone analogue of (I). As expected, the oxidation destroys the aromaticity of the six-carbon ring, reflected in a shortening of C2—C3 and C5—C6 and a lengthening of the other ring C—C bonds (Allen *et al.*, 1987 ▸).
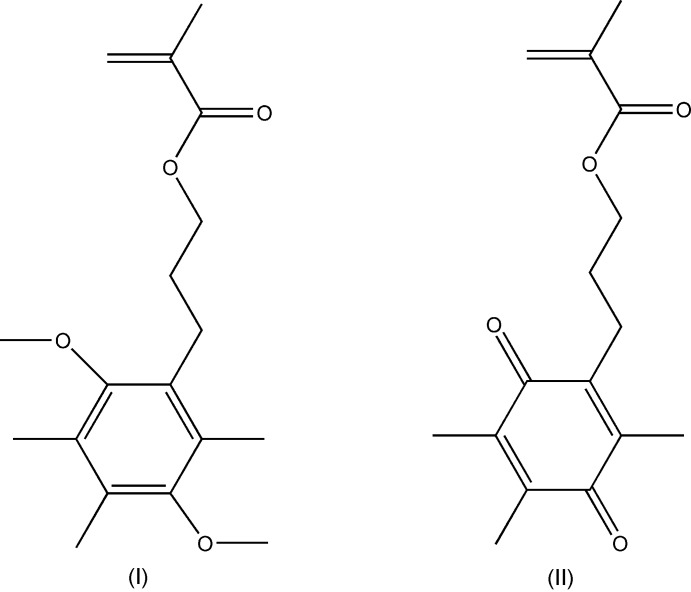


The spatial arrangement of the ring substituents and the propyl methacrylate moiety is remarkably similar to that observed for (I). In particular, the torsional geometry of the vinyl and carbonyl components of the methacrylate groups of both (I) and (II) display the typical *s-trans* preference (McAdam *et al.*, 2015 ▸). Predictably, both the benzene and quinone ring systems (C1–C6) and the attached atoms (O1, C21, C31, O4, C51 and C7) are nearly planar, with r.m.s. deviations from the mean planes of 0.0377 and 0.0158 Å, respectively.

## Supra­molecular features   

### Crystal packing for (I)   

**Figure 3:**
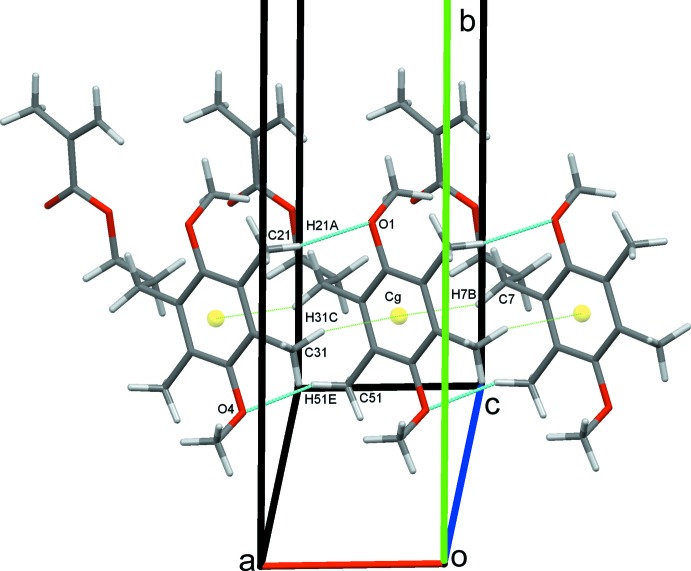
Chains of mol­ecules of (I) along the *a-*axis direction.

**Figure 4:**
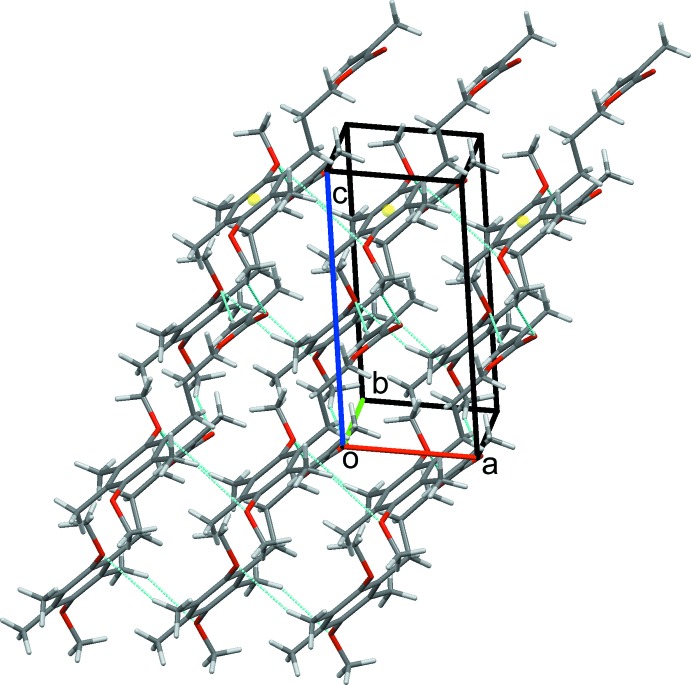
A double sheet of mol­ecules of (I) in the *ac* plane.

**Figure 5:**
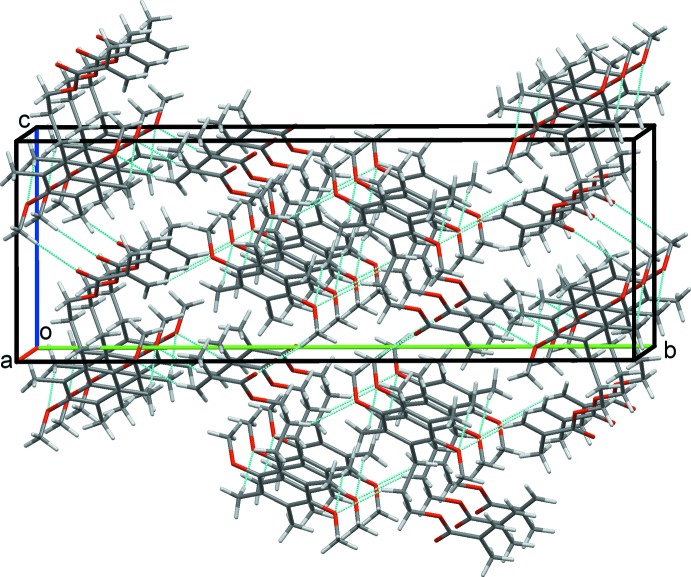
Overall packing for (I) viewed along the *a-*axis direction.

In the crystal structure of (I), C21—H21*A*⋯O1 and C51—H51*E*⋯O4 hydrogen bonds form chains of mol­ecules along the *a*-axis direction. The chains are reinforced by C7—H7*B*⋯*Cg* and C31—H31*C*⋯*Cg* contacts (*Cg* is the centroid of the C1–C6 ring) between methyl and methyl­ene group hydrogen atoms and the aromatic ring, Table 1 ▸ and Fig. 3 ▸. C12—H12*B*⋯O1 and C41—H41*A*⋯O10, hydrogen bonds link these chains into a sheet, two-mol­ecules thick, that lies parallel to the *ac* plane (010), Fig. 4 ▸. Extension to a three-dimensional structure is completed by C8—H8*B*⋯O4 inversion dimers. These form 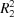
(14) rings and link pairs of double-layer sheets, stacking mol­ecules along the *a*-axis direction, Fig. 5 ▸.

### Crystal packing for (II)   

**Figure 6:**
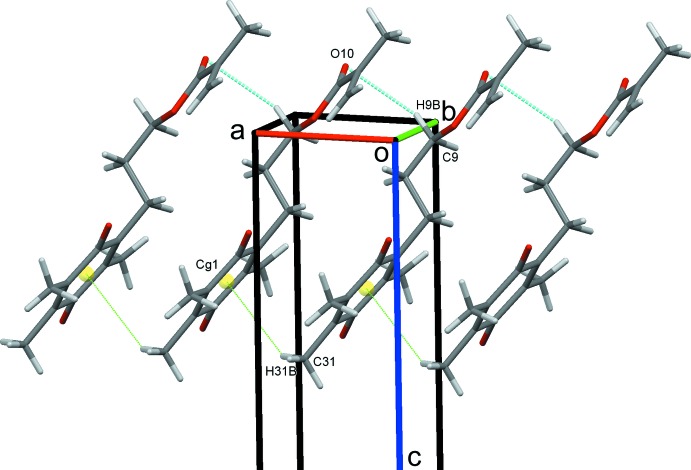
Chains of mol­ecules of (II) formed along *a*.

**Figure 7:**
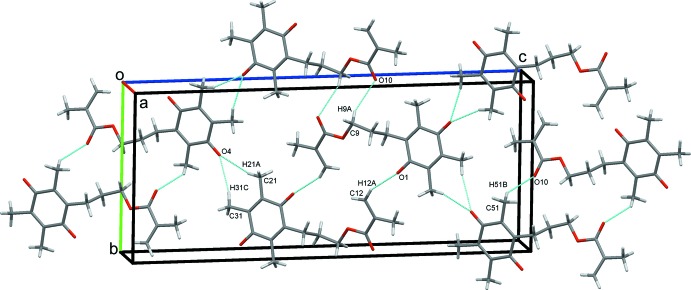
Sheets of mol­ecules of (II) viewed along *a*.

**Figure 8:**
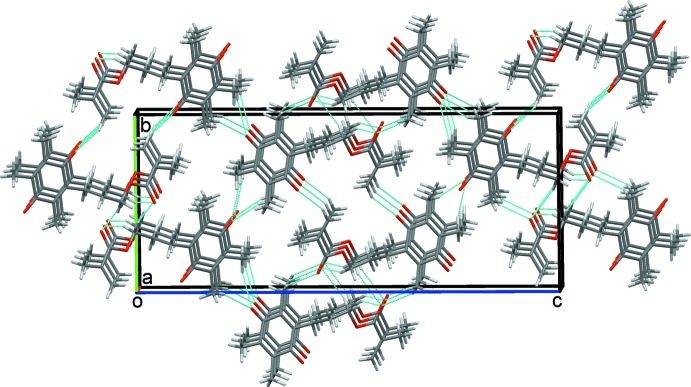
Overall packing for (II) viewed along the *a*-axis direction.

For (II), an extensive series of C—H⋯O hydrogen bonds and a C—H⋯π(ring) contact generate the three-dimensional structure. These contacts include O10 acting as a trifurcated acceptor; C9—H9*B*⋯O10 hydrogen bonds, supported by C31—H31*B*⋯*Cg* contacts (*Cg* is the centroid of the C1–C6 ring),Table 2 ▸, form chains along the *a-*axis direction, Fig. 6 ▸. The other two components of the trifurcate, the inversion-related C9—H9*A*⋯O10 and C51—H51*B*⋯O10 hydrogen bonds form 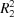
(10) and 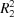
(20) rings, respectively. A third inversion dimer results from C12—H12*A*⋯O1 contacts and forms 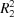
(22) rings. O4 acts as a bifurcated acceptor, forming C21—H21*A*⋯O4 and C31—H31*C*⋯O4 hydrogen bonds that enclose 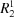
(7) rings, completing an extensive sheet of mol­ecules parallel to (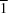
05), Fig. 7 ▸. This eclectic array of contacts combine to produce a three-dimensional network with mol­ecules stacked along the *a* axis, Fig. 8 ▸.

## Database survey   

A search of the CSD (Version 5.37 November 2015 with three updates; Groom *et al.*, 2016 ▸) revealed a surprising degree of exclusivity for both of the title compounds. A search for the 2,5-dimeth­oxy-3,4,6-tri­methyl­phenyl segment of (I) produced only two hits, our earlier report of the precursor 2,5-dimeth­oxy-3,4,6-tri­methyl­benzaldehyde (Wickramasinhage *et al.*, 2016 ▸) and the dimer bis­(2,5-dimeth­oxy-3,4,6-tri­methyl­phen­yl)methane (Wiedenfeld *et al.*, 2003 ▸). A search for the corresponding quinone ring system was even less productive, with octa­methyl-1,4-cyclo­hexa­nedione the only related structure (Hoffmann & Hursthouse, 1976 ▸). Structures containing the propyl methacrylate moiety were similarly scarce, with the fullerene derivative 4-(6,9,12,15,18-penta­methyl-C_60_fulleren-1-yl)butyl methacrylate di­chloro­methane solvate (Matsuo *et al.*, 2009 ▸) and a tungsten polyphosphate derivative (Hasegawa *et al.*, 2007 ▸) the only hits.

## Synthesis and crystallization   

**Figure 9:**
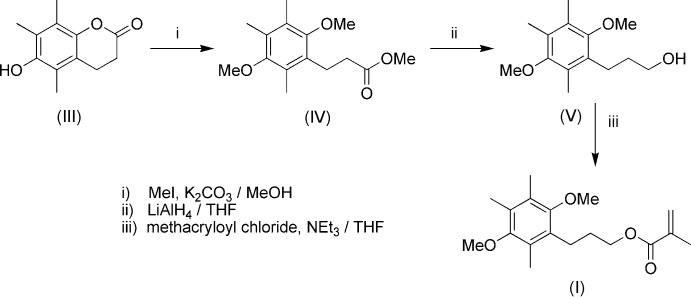
Steps involved in the synthesis of compound (I).

The synthesis of (I) was accomplished in three steps (Fig. 9 ▸) from 6-hy­droxy-5,7,8-tri­methyl­chroman-2-one (III) (Goswami *et al.*, 2011 ▸) as described below.

**Methyl­ation of 6-hy­droxy-5,7,8-tri­methyl­chroman-2-one (III):** To a solution of (III) (5 g, 24 mmol) and dry K_2_CO_3_ (13.4 g, 97 mmol) in MeOH (50 mL) was added MeI (13.8 mL, 97 mmol). The mixture was refluxed for 4 h, filtered through celite, and solvent removed *in vacuo* to afford methyl 3-(2,5-dimeth­oxy-3,4,6-tri­methyl­phen­yl)propano­ate (IV) (5.5 g, 85%) as a yellow liquid. MS calculated for [C_15_H_22_NaO_4_]^+^: 289.1410. Found: 289.1391 (6.72 ppm). IR (KBr) ν_C=O_: 1750 cm^−1^ (methyl ester). ^1^H NMR (CDCl_3_, δ ppm): 2.18 (*s*, 6H, 2 × Ar–CH_3_), 2.24 (*s*, 3H, Ar-CH_3_), 2.49 & 2.95 [2 × (*t*, *J* = 7.8 Hz, 2H, CH_2_)], 3.65 (*s*, 3H, ester OCH_3_), 3.68 & 3.71 [2 × (*s*, 3H, Ar–OCH_3_)]. ^13^C NMR (CDCl_3_, δ ppm): 12.3, 12.8, 13.0, 23.0, 34.5, 51.5, 60.6, 61.1, 127.5, 128.4, 129.2, 130.4, 153.3, 174.0.

**Reduction of methyl 3-(2,5-dimeth­oxy-3,4,6-tri­methyl­phen­yl)propano­ate (IV):** To a stirred suspension of 0.85 g (22 mmol) of LiAlH_4_ in 100 mL dry THF cooled to 273 K in ice a solution of 5.0 g (18.7 mmol) of (IV) in 100 mL THF was added dropwise. After the vigorous reaction subsided, the mixture was heated to reflux for 2 h. Excess of the hydride was decomposed by careful addition of water, and the mixture was neutralized with acetic acid. To this was added 650 mL of saturated aq. NH_4_Cl solution. The organic layer was separated and the aqueous layer further extracted with 4 × 150 mL portions of THF. The combined THF layers were dried over MgSO_4_ and solvent removed *in vacuo*. Recrystallization from Et_2_O gave 4.1 mg (91%) of 3-(2,5-dimeth­oxy-3,4,6-tri­methyl­phen­yl)propan-1-ol (V) as a white solid, m.p. 461–463 K please check. MS calculated for C_14_H_22_NaO_3_]^+^: 261.1461. Found: 246.1461 (0 ppm). IR (KBr) ν_OH_: 3425, 3150 cm^−1^. ^1^H NMR (CDCl_3_, δ ppm): 1.75 (*m*, 2H, CH_2_), 2.09 (*s*, 1H OH), 2.18 (*s*, 6H, 2 × Ar–CH_3_), 2.23 (*s*, 3H, Ar–CH_3_), 2.75 (*t*, *J* = 7.3 Hz, 2H, Ar–CH_2_), 3.52 (*t*, *J* = 6.6 Hz, 2H, CH_2_–OH), 3.65 & 3.69 [2 × (*s*, 3H, Ar–OCH_3_)]. ^13^C NMR (CDCl_3_, δ ppm): 11.7, 12.6, 12.8, 22.6, 32.0, 60.0, 61.0, 61.2, 127.4, 127.6, 128.6, 129.2, 130.4, 153.0, 153.5.

**Acyl­ation of 3-(2,5-dimeth­oxy-3,4,6-tri­methyl­phen­yl)propan-1-ol (V):** The alcohol (V) (5.0 g, 21 mmol) was dissolved in CH_2_Cl_2_ (100 ml). NEt_3_ (2.2 mL) was added and the solution stirred 30 min at 273 K. Methacryloyl chloride (2.4 g, 23 mmol) was added dropwise, stirred for 2 h under nitro­gen at 273 K and then at room temperature for 4 h. After extraction from CH_2_Cl_2_/H_2_O the organic layer was dried (MgSO_4_) and solvent removed *in vacuo*. Purification using chromatography on SiO_2_ using petroleum ether/EtOAc (9:1) gave the colourless solid product (I), m.p. 395–397 K. MS calculated for [C_18_H_26_NaO_4_]^+^: 329.1723. Found: 329.1709 (4.41 ppm). IR (KBr) ν_C=O_: 1731 cm^−1^ (ester). ^1^H NMR (CDCl_3_, δ ppm): 1.89 (*m*, 2H, CH_2_), 1.98 (*m*, 3H, CH_3_), 2.19 (*s*, 6H, 2 × Ar–CH_3_), 2.23 (*s*, 3H, Ar–CH_3_), 2.73 (*t*, *J* = 7.6 Hz, 2H, Ar–CH_2_), 3.65 & 3.68 [2 × (*s*, 3H, Ar–OCH_3_)], 4.23 (*t*, *J* = 6.1 Hz, 2H, CH_2_), 5.57 (*m*, 1H, =CH), 6.14 (*m*, 1H, =CH). ^13^C NMR (CDCl_3_, δ ppm): 12.2, 12.9, 13.1, 18.6, 24.1, 29.5, 60.3, 61.1, 65.0, 125.4, 127.4, 128.2, 128.8, 131.4, 136.8, 153.20, 153.4, 167.8. Crystals of (I) were obtained from a mixed CH_2_Cl_2_/hexane solution 1/1 *v*/*v*.

The synthesis of (II) has been reported previously (Goswami *et al.*, 2013 ▸). Crystals were obtained from the slow evaporation of an Et_2_O solution.

## Refinement   

Crystal data, data collection and structure refinement details are summarized in Table 3 ▸. All H atoms were refined using a riding model with *d*(C—H) = 0.95 Å, *U*_iso_ = 1.2*U*_eq_(C) for vinyl, 0.99 Å, *U*_iso_ = 1.2*U*_eq_(C) for CH_2_ H atoms and 0.98 Å, *U*_iso_ = 1.5*U*_eq_(C) for CH_3_ H atoms. The hydrogen atoms of the C13 and C51 methyl groups of (I) were equally disordered over two sites. Idealized disorder models were applied using AFIX123 in *SHELXL2014/7*. For (I), a low-angle reflection with *F*_o_ << *F*_c_, that may have been affected by the beam-stop, was omitted from the final refinement cycles.

## Supplementary Material

Crystal structure: contains datablock(s) global, I, II. DOI: 10.1107/S2056989017004959/hg5486sup1.cif

Structure factors: contains datablock(s) I. DOI: 10.1107/S2056989017004959/hg5486Isup2.hkl

Structure factors: contains datablock(s) II. DOI: 10.1107/S2056989017004959/hg5486IIsup3.hkl

Supporting information file. DOI: 10.1107/S2056989017004959/hg5486Isup4.cml

Supporting information file. DOI: 10.1107/S2056989017004959/hg5486IIsup5.cml

CCDC references: 1541065, 1541064

Additional supporting information:  crystallographic information; 3D view; checkCIF report

## Figures and Tables

**Table 1:** Hydrogen-bond geometry (Å, °) for (I) *Cg* is the centroid of the C1–C6 benzene ring

*D*—H⋯*A*	*D*—H	H⋯*A*	*D*⋯*A*	*D*—H⋯*A*
C8—H8*B*⋯O4^i^	0.99	2.58	3.456 (4)	147
C12—H12*B*⋯O1^ii^	0.95	2.50	3.388 (4)	157
C21—H21*A*⋯O1^iii^	0.98	2.67	3.614 (5)	161
C41—H41*A*⋯O10^iv^	0.98	2.66	3.590 (5)	159
C51—H51*E*⋯O4^v^	0.98	2.65	3.541 (4)	151
C7—H7*B*⋯*Cg* ^v^	0.99	2.97	3.709 (4)	134
C31—H31*C*⋯*Cg* ^iii^	0.98	2.85	3.693 (4)	148

Symmetry codes: (i) 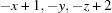; (ii) 
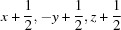
; (iii) 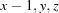; (iv) 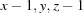; (v) 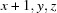.

**Table 2:** Hydrogen-bond geometry (Å, °) for (II) *Cg* is the centroid of the C1–C6 ring.

*D*—H⋯*A*	*D*—H	H⋯*A*	*D*⋯*A*	*D*—H⋯*A*
C9—H9*A*⋯O10^i^	0.99	2.72	3.624 (3)	153
C9—H9*B*⋯O10^ii^	0.99	2.70	3.595 (3)	150
C12—H12*A*⋯O1^iii^	0.95	2.53	3.422 (3)	156
C21—H21*A*⋯O4^iv^	0.98	2.52	3.455 (3)	161
C31—H31*C*⋯O4^iv^	0.98	2.68	3.638 (3)	166
C51—H51*B*⋯O10^v^	0.98	2.67	3.510 (3)	144
C31—H31*B*⋯*Cg* ^ii^	0.98	2.95	3.534 (3)	119

Symmetry codes: (i) 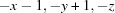; (ii) 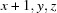; (iii) 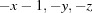; (iv) 
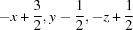
; (v) 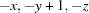.

**Table 3:** Experimental details

	(I)	(II)
Crystal data		
Chemical formula	C_18_H_26_O_4_	C_16_H_20_O_4_
*M*_r_	306.39	276.32
Crystal system, space group	Monoclinic, *P*2_1_/*n*	Monoclinic, *P*2_1_/*n*
Temperature (K)	91	89
*a*, *b*, *c* (Å)	5.1833 (7), 30.341 (4), 10.6339 (15)	4.4096 (2), 11.8425 (6), 28.2511 (16)
β (°)	97.910 (9)	93.495 (3)
*V* (Å^3^)	1656.4 (4)	1472.55 (13)
*Z*	4	4
Radiation type	Mo *K*α	Mo *K*α
μ (mm^−1^)	0.09	0.09
Crystal size (mm)	0.65 × 0.04 × 0.04	0.27 × 0.14 × 0.13

Data collection		
Diffractometer	Bruker APEXII CCD area detector	Bruker APEXII CCD area detector
Absorption correction	Multi-scan (*SADABS*; Bruker, 2013 ▸)	Multi-scan (*SADABS*; Bruker, 2013 ▸)
*T*_min_, *T*_max_	0.775, 1.00	0.785, 1.000
No. of measured, independent and observed [*I* > 2σ(*I*)] reflections	11585, 1681, 1254	16398, 2509, 1774
*R*_int_	0.081	0.071
θ_max_ (°)	20.7	24.8
(sin θ/λ)_max_ (Å^−1^)	0.497	0.591

Refinement		
*R*[*F* ^2^ > 2σ(*F* ^2^)], *wR*(*F* ^2^), *S*	0.052, 0.138, 1.03	0.049, 0.138, 1.04
No. of reflections	1681	2509
No. of parameters	203	185
H-atom treatment	H-atom parameters constrained	H-atom parameters constrained
Δρ_max_, Δρ_min_ (e Å^−3^)	0.34, −0.24	0.34, −0.32

Computer programs: *APEX2* and *SAINT* (Bruker, 2013 ▸), *SHELXS2013* (Sheldrick, 2008 ▸), *SHELXL2014/7* (Sheldrick, 2015 ▸), *TITAN* (Hunter & Simpson, 1999 ▸), *Mercury* (Macrae *et al.*, 2008 ▸), *enCIFer* (Allen *et al.*, 2004 ▸), *PLATON* (Spek, 2009 ▸) and *publCIF* (Westrip 2010 ▸).

## supplementary crystallographic information

### (I) 3-(2,5-Dimethoxy-3,4,6-trimethylphenyl)propyl methacrylate Crystal data

**Table:**

C_18_H_26_O_4_	*F*(000) = 664
*M**_r_* = 306.39	*D*_x_ = 1.229 Mg m^−^^3^
Monoclinic, *P*2_1_/*n*	Mo *K*α radiation, λ = 0.71073 Å
*a* = 5.1833 (7) Å	Cell parameters from 1289 reflections
*b* = 30.341 (4) Å	θ = 4.7–40.3°
*c* = 10.6339 (15) Å	µ = 0.09 mm^−^^1^
β = 97.910 (9)°	*T* = 91 K
*V* = 1656.4 (4) Å^3^	Needle, colourless
*Z* = 4	0.65 × 0.04 × 0.04 mm

### (I) 3-(2,5-Dimethoxy-3,4,6-trimethylphenyl)propyl methacrylate Data collection

**Table:**

Bruker APEXII CCD area detector diffractometer	1681 independent reflections
Radiation source: fine-focus sealed tube	1254 reflections with *I* > 2σ(*I*)
Graphite monochromator	*R*_int_ = 0.081
ω scans	θ_max_ = 20.7°, θ_min_ = 2.4°
Absorption correction: multi-scan (SADABS; Bruker, 2013)	*h* = −5→5
*T*_min_ = 0.775, *T*_max_ = 1.00	*k* = −29→29
11585 measured reflections	*l* = −10→10

### (I) 3-(2,5-Dimethoxy-3,4,6-trimethylphenyl)propyl methacrylate Refinement

**Table:**

Refinement on *F*^2^	0 restraints
Least-squares matrix: full	Hydrogen site location: inferred from neighbouring sites
*R*[*F*^2^ > 2σ(*F*^2^)] = 0.052	H-atom parameters constrained
*wR*(*F*^2^) = 0.138	*w* = 1/[σ^2^(*F*_o_^2^) + (0.0683*P*)^2^ + 1.3102*P*] where *P* = (*F*_o_^2^ + 2*F*_c_^2^)/3
*S* = 1.03	(Δ/σ)_max_ < 0.001
1681 reflections	Δρ_max_ = 0.34 e Å^−^^3^
203 parameters	Δρ_min_ = −0.24 e Å^−^^3^

### (I) 3-(2,5-Dimethoxy-3,4,6-trimethylphenyl)propyl methacrylate Special details

**Table:**

Geometry. All esds (except the esd in the dihedral angle between two l.s. planes) are estimated using the full covariance matrix. The cell esds are taken into account individually in the estimation of esds in distances, angles and torsion angles; correlations between esds in cell parameters are only used when they are defined by crystal symmetry. An approximate (isotropic) treatment of cell esds is used for estimating esds involving l.s. planes.
Refinement. One low angle reflection with Fo << Fc, that may have been affected by the beam-stop, was omitted from the final refinement cycles.

### (I) 3-(2,5-Dimethoxy-3,4,6-trimethylphenyl)propyl methacrylate Fractional atomic coordinates and isotropic or equivalent isotropic displacement parameters (Å^2^)

**Table:**

	*x*	*y*	*z*	*U*_iso_*/*U*_eq_	Occ. (<1)
C1	0.5070 (7)	0.13988 (12)	0.9190 (3)	0.0223 (10)	
O1	0.6011 (5)	0.17928 (8)	0.9780 (2)	0.0255 (7)	
C111	0.4922 (8)	0.18964 (12)	1.0910 (4)	0.0324 (11)	
H11A	0.3020	0.1872	1.0741	0.049*	
H11B	0.5403	0.2198	1.1176	0.049*	
H11C	0.5595	0.1690	1.1585	0.049*	
C2	0.2886 (7)	0.14223 (11)	0.8270 (3)	0.0213 (10)	
C21	0.1425 (7)	0.18507 (12)	0.7978 (4)	0.0296 (10)	
H21A	−0.0289	0.1831	0.8265	0.044*	
H21B	0.1204	0.1904	0.7061	0.044*	
H21C	0.2414	0.2094	0.8420	0.044*	
C3	0.2007 (7)	0.10363 (12)	0.7625 (3)	0.0198 (10)	
C31	−0.0362 (7)	0.10426 (12)	0.6621 (3)	0.0263 (10)	
H31A	−0.0762	0.0742	0.6321	0.039*	
H31B	−0.0008	0.1227	0.5908	0.039*	
H31C	−0.1850	0.1163	0.6984	0.039*	
C4	0.3397 (7)	0.06474 (11)	0.7918 (3)	0.0189 (10)	
O4	0.2485 (5)	0.02580 (7)	0.7301 (2)	0.0235 (7)	
C41	0.3680 (8)	0.01709 (13)	0.6187 (3)	0.0309 (11)	
H41A	0.3442	0.0426	0.5617	0.046*	
H41B	0.2869	−0.0089	0.5750	0.046*	
H41C	0.5545	0.0116	0.6432	0.046*	
C5	0.5615 (7)	0.06238 (11)	0.8830 (3)	0.0185 (9)	
C51	0.7126 (7)	0.01998 (11)	0.9032 (3)	0.0240 (10)	
H51A	0.8608	0.0242	0.9700	0.036*	0.5
H51B	0.5991	−0.0032	0.9287	0.036*	0.5
H51C	0.7763	0.0114	0.8241	0.036*	0.5
H51D	0.6300	−0.0026	0.8452	0.036*	0.5
H51E	0.8918	0.0248	0.8865	0.036*	0.5
H51F	0.7145	0.0101	0.9911	0.036*	0.5
C6	0.6460 (7)	0.10094 (12)	0.9490 (3)	0.0211 (10)	
C7	0.8823 (7)	0.10066 (11)	1.0496 (3)	0.0229 (10)	
H7A	0.9480	0.1312	1.0626	0.028*	
H7B	1.0210	0.0829	1.0188	0.028*	
C8	0.8279 (7)	0.08205 (12)	1.1773 (3)	0.0233 (9)	
H8A	0.6784	0.0980	1.2044	0.028*	
H8B	0.7780	0.0507	1.1660	0.028*	
C9	1.0568 (7)	0.08561 (12)	1.2801 (3)	0.0268 (10)	
H9A	1.0197	0.0700	1.3574	0.032*	
H9B	1.2130	0.0722	1.2517	0.032*	
O9	1.1017 (5)	0.13212 (8)	1.3071 (2)	0.0266 (7)	
C10	1.3096 (8)	0.14204 (13)	1.3916 (4)	0.0243 (10)	
O10	1.4618 (5)	0.11468 (9)	1.4395 (2)	0.0335 (8)	
C11	1.3351 (7)	0.19019 (12)	1.4190 (3)	0.0248 (10)	
C12	1.1719 (8)	0.21884 (13)	1.3569 (4)	0.0330 (11)	
H12A	1.0364	0.2088	1.2941	0.040*	
H12B	1.1902	0.2494	1.3753	0.040*	
C13	1.5513 (8)	0.20272 (14)	1.5203 (4)	0.0391 (11)	
H13A	1.6460	0.1762	1.5525	0.059*	0.5
H13B	1.6707	0.2228	1.4850	0.059*	0.5
H13C	1.4791	0.2174	1.5898	0.059*	0.5
H13D	1.5512	0.2347	1.5324	0.059*	0.5
H13E	1.5265	0.1881	1.5999	0.059*	0.5
H13F	1.7181	0.1936	1.4951	0.059*	0.5

### (I) 3-(2,5-Dimethoxy-3,4,6-trimethylphenyl)propyl methacrylate Atomic displacement parameters (Å^2^)

**Table:**

	*U*^11^	*U*^22^	*U*^33^	*U*^12^	*U*^13^	*U*^23^
C1	0.027 (2)	0.023 (2)	0.020 (2)	−0.006 (2)	0.012 (2)	−0.0020 (18)
O1	0.0321 (16)	0.0217 (16)	0.0244 (16)	−0.0032 (12)	0.0100 (13)	−0.0026 (12)
C111	0.042 (3)	0.030 (3)	0.028 (3)	0.002 (2)	0.013 (2)	−0.0046 (19)
C2	0.022 (2)	0.022 (2)	0.022 (2)	0.0017 (19)	0.010 (2)	0.0053 (18)
C21	0.025 (2)	0.030 (2)	0.034 (2)	0.0009 (19)	0.0036 (19)	0.0006 (19)
C3	0.022 (2)	0.026 (2)	0.013 (2)	−0.0025 (19)	0.0075 (18)	−0.0001 (18)
C31	0.031 (3)	0.027 (2)	0.022 (2)	−0.0015 (19)	0.007 (2)	0.0011 (17)
C4	0.026 (2)	0.017 (2)	0.015 (2)	−0.0043 (19)	0.009 (2)	−0.0017 (17)
O4	0.0286 (15)	0.0231 (15)	0.0205 (16)	−0.0024 (13)	0.0097 (12)	−0.0029 (12)
C41	0.039 (3)	0.032 (3)	0.024 (3)	−0.003 (2)	0.014 (2)	−0.0054 (19)
C5	0.017 (2)	0.020 (2)	0.020 (2)	0.0001 (18)	0.0082 (19)	0.0013 (18)
C51	0.025 (2)	0.024 (2)	0.025 (2)	−0.0010 (18)	0.0067 (18)	−0.0010 (17)
C6	0.022 (2)	0.024 (2)	0.019 (2)	−0.0029 (19)	0.0097 (19)	0.0001 (18)
C7	0.026 (2)	0.020 (2)	0.023 (2)	0.0012 (18)	0.0058 (19)	0.0003 (17)
C8	0.026 (2)	0.023 (2)	0.021 (2)	−0.0020 (19)	0.0056 (18)	0.0022 (18)
C9	0.034 (2)	0.022 (2)	0.025 (2)	−0.004 (2)	0.0046 (19)	−0.0003 (19)
O9	0.0300 (17)	0.0213 (16)	0.0274 (16)	−0.0022 (12)	0.0001 (14)	−0.0027 (12)
C10	0.024 (3)	0.033 (3)	0.018 (2)	−0.002 (2)	0.011 (2)	−0.002 (2)
O10	0.0331 (17)	0.0316 (17)	0.0347 (18)	0.0013 (15)	0.0005 (14)	0.0017 (14)
C11	0.025 (2)	0.027 (3)	0.024 (2)	0.001 (2)	0.009 (2)	−0.0012 (19)
C12	0.036 (3)	0.026 (3)	0.037 (3)	−0.006 (2)	0.008 (2)	−0.011 (2)
C13	0.039 (3)	0.037 (3)	0.041 (3)	−0.008 (2)	0.005 (2)	−0.007 (2)

### (I) 3-(2,5-Dimethoxy-3,4,6-trimethylphenyl)propyl methacrylate Geometric parameters (Å, º)

**Table:**

C1—C2	1.393 (5)	C51—H51C	0.9800
C1—C6	1.398 (5)	C51—H51D	0.9800
C1—O1	1.406 (4)	C51—H51E	0.9800
O1—C111	1.431 (4)	C51—H51F	0.9800
C111—H11A	0.9800	C6—C7	1.512 (5)
C111—H11B	0.9800	C7—C8	1.532 (5)
C111—H11C	0.9800	C7—H7A	0.9900
C2—C3	1.401 (5)	C7—H7B	0.9900
C2—C21	1.515 (5)	C8—C9	1.503 (5)
C21—H21A	0.9800	C8—H8A	0.9900
C21—H21B	0.9800	C8—H8B	0.9900
C21—H21C	0.9800	C9—O9	1.452 (4)
C3—C4	1.395 (5)	C9—H9A	0.9900
C3—C31	1.512 (5)	C9—H9B	0.9900
C31—H31A	0.9800	O9—C10	1.339 (4)
C31—H31B	0.9800	C10—O10	1.209 (4)
C31—H31C	0.9800	C10—C11	1.492 (5)
C4—C5	1.400 (5)	C11—C12	1.324 (5)
C4—O4	1.402 (4)	C11—C13	1.493 (5)
O4—C41	1.435 (4)	C12—H12A	0.9500
C41—H41A	0.9800	C12—H12B	0.9500
C41—H41B	0.9800	C13—H13A	0.9800
C41—H41C	0.9800	C13—H13B	0.9800
C5—C6	1.403 (5)	C13—H13C	0.9800
C5—C51	1.506 (5)	C13—H13D	0.9800
C51—H51A	0.9800	C13—H13E	0.9800
C51—H51B	0.9800	C13—H13F	0.9800
			
C2—C1—C6	123.2 (3)	H51A—C51—H51F	56.3
C2—C1—O1	118.0 (3)	H51B—C51—H51F	56.3
C6—C1—O1	118.7 (3)	H51C—C51—H51F	141.1
C1—O1—C111	114.2 (3)	H51D—C51—H51F	109.5
O1—C111—H11A	109.5	H51E—C51—H51F	109.5
O1—C111—H11B	109.5	C1—C6—C5	118.4 (3)
H11A—C111—H11B	109.5	C1—C6—C7	120.5 (3)
O1—C111—H11C	109.5	C5—C6—C7	121.1 (3)
H11A—C111—H11C	109.5	C6—C7—C8	113.6 (3)
H11B—C111—H11C	109.5	C6—C7—H7A	108.9
C1—C2—C3	118.6 (3)	C8—C7—H7A	108.9
C1—C2—C21	121.5 (3)	C6—C7—H7B	108.9
C3—C2—C21	119.8 (3)	C8—C7—H7B	108.9
C2—C21—H21A	109.5	H7A—C7—H7B	107.7
C2—C21—H21B	109.5	C9—C8—C7	113.3 (3)
H21A—C21—H21B	109.5	C9—C8—H8A	108.9
C2—C21—H21C	109.5	C7—C8—H8A	108.9
H21A—C21—H21C	109.5	C9—C8—H8B	108.9
H21B—C21—H21C	109.5	C7—C8—H8B	108.9
C4—C3—C2	118.3 (3)	H8A—C8—H8B	107.7
C4—C3—C31	120.8 (3)	O9—C9—C8	107.6 (3)
C2—C3—C31	120.8 (3)	O9—C9—H9A	110.2
C3—C31—H31A	109.5	C8—C9—H9A	110.2
C3—C31—H31B	109.5	O9—C9—H9B	110.2
H31A—C31—H31B	109.5	C8—C9—H9B	110.2
C3—C31—H31C	109.5	H9A—C9—H9B	108.5
H31A—C31—H31C	109.5	C10—O9—C9	116.3 (3)
H31B—C31—H31C	109.5	O10—C10—O9	123.1 (3)
C3—C4—C5	123.2 (3)	O10—C10—C11	123.7 (4)
C3—C4—O4	118.5 (3)	O9—C10—C11	113.1 (3)
C5—C4—O4	118.2 (3)	C12—C11—C10	120.8 (4)
C4—O4—C41	112.7 (3)	C12—C11—C13	123.8 (4)
O4—C41—H41A	109.5	C10—C11—C13	115.3 (3)
O4—C41—H41B	109.5	C11—C12—H12A	120.0
H41A—C41—H41B	109.5	C11—C12—H12B	120.0
O4—C41—H41C	109.5	H12A—C12—H12B	120.0
H41A—C41—H41C	109.5	C11—C13—H13A	109.5
H41B—C41—H41C	109.5	C11—C13—H13B	109.5
C4—C5—C6	118.3 (3)	H13A—C13—H13B	109.5
C4—C5—C51	120.3 (3)	C11—C13—H13C	109.5
C6—C5—C51	121.3 (3)	H13A—C13—H13C	109.5
C5—C51—H51A	109.5	H13B—C13—H13C	109.5
C5—C51—H51B	109.5	C11—C13—H13D	109.5
H51A—C51—H51B	109.5	H13A—C13—H13D	141.1
C5—C51—H51C	109.5	H13B—C13—H13D	56.3
H51A—C51—H51C	109.5	H13C—C13—H13D	56.3
H51B—C51—H51C	109.5	C11—C13—H13E	109.5
C5—C51—H51D	109.5	H13A—C13—H13E	56.3
H51A—C51—H51D	141.1	H13B—C13—H13E	141.1
H51B—C51—H51D	56.3	H13C—C13—H13E	56.3
H51C—C51—H51D	56.3	H13D—C13—H13E	109.5
C5—C51—H51E	109.5	C11—C13—H13F	109.5
H51A—C51—H51E	56.3	H13A—C13—H13F	56.3
H51B—C51—H51E	141.1	H13B—C13—H13F	56.3
H51C—C51—H51E	56.3	H13C—C13—H13F	141.1
H51D—C51—H51E	109.5	H13D—C13—H13F	109.5
C5—C51—H51F	109.5	H13E—C13—H13F	109.5
			
C2—C1—O1—C111	90.7 (4)	C2—C1—C6—C5	0.1 (5)
C6—C1—O1—C111	−93.5 (4)	O1—C1—C6—C5	−175.5 (3)
C6—C1—C2—C3	1.0 (5)	C2—C1—C6—C7	179.8 (3)
O1—C1—C2—C3	176.7 (3)	O1—C1—C6—C7	4.2 (5)
C6—C1—C2—C21	179.6 (3)	C4—C5—C6—C1	−1.0 (5)
O1—C1—C2—C21	−4.7 (5)	C51—C5—C6—C1	175.4 (3)
C1—C2—C3—C4	−1.3 (5)	C4—C5—C6—C7	179.3 (3)
C21—C2—C3—C4	−179.9 (3)	C51—C5—C6—C7	−4.2 (5)
C1—C2—C3—C31	179.7 (3)	C1—C6—C7—C8	101.6 (4)
C21—C2—C3—C31	1.1 (5)	C5—C6—C7—C8	−78.7 (4)
C2—C3—C4—C5	0.4 (5)	C6—C7—C8—C9	−174.8 (3)
C31—C3—C4—C5	179.4 (3)	C7—C8—C9—O9	66.7 (4)
C2—C3—C4—O4	178.3 (3)	C8—C9—O9—C10	−176.0 (3)
C31—C3—C4—O4	−2.7 (5)	C9—O9—C10—O10	3.5 (5)
C3—C4—O4—C41	93.9 (4)	C9—O9—C10—C11	−177.2 (3)
C5—C4—O4—C41	−88.1 (4)	O10—C10—C11—C12	175.3 (4)
C3—C4—C5—C6	0.8 (5)	O9—C10—C11—C12	−4.0 (5)
O4—C4—C5—C6	−177.1 (3)	O10—C10—C11—C13	−5.4 (5)
C3—C4—C5—C51	−175.7 (3)	O9—C10—C11—C13	175.3 (3)
O4—C4—C5—C51	6.4 (5)		

### (I) 3-(2,5-Dimethoxy-3,4,6-trimethylphenyl)propyl methacrylate Hydrogen-bond geometry (Å, º)

Cg is the centroid of the C1–C6 benzene ring

**Table:**

*D*—H···*A*	*D*—H	H···*A*	*D*···*A*	*D*—H···*A*
C8—H8*B*···O4^i^	0.99	2.58	3.456 (4)	147
C12—H12*B*···O1^ii^	0.95	2.50	3.388 (4)	157
C21—H21*A*···O1^iii^	0.98	2.67	3.614 (5)	161
C41—H41*A*···O10^iv^	0.98	2.66	3.590 (5)	159
C51—H51*E*···O4^v^	0.98	2.65	3.541 (4)	151
C7—H7*B*···*Cg*^v^	0.99	2.97	3.709 (4)	134
C31—H31*C*···*Cg*^iii^	0.98	2.85	3.693 (4)	148

Symmetry codes: (i) −*x*+1, −*y*, −*z*+2; (ii) *x*+1/2, −*y*+1/2, *z*+1/2; (iii) *x*−1, *y*, *z*; (iv) *x*−1, *y*, *z*−1; (v) *x*+1, *y*, *z*.

### (II) 3-(2,4,5-Trimethyl-3,6-dioxocyclohexa-1,4-dienyl)propyl methacrylate Crystal data

**Table:**

C_16_H_20_O_4_	*F*(000) = 592
*M**_r_* = 276.32	*D*_x_ = 1.246 Mg m^−^^3^
Monoclinic, *P*2_1_/*n*	Mo *K*α radiation, λ = 0.71073 Å
*a* = 4.4096 (2) Å	Cell parameters from 1562 reflections
*b* = 11.8425 (6) Å	θ = 2.3–21.1°
*c* = 28.2511 (16) Å	µ = 0.09 mm^−^^1^
β = 93.495 (3)°	*T* = 89 K
*V* = 1472.55 (13) Å^3^	Irregular fragment, colourless
*Z* = 4	0.27 × 0.14 × 0.13 mm

### (II) 3-(2,4,5-Trimethyl-3,6-dioxocyclohexa-1,4-dienyl)propyl methacrylate Data collection

**Table:**

Bruker APEXII CCD area detector diffractometer	1774 reflections with *I* > 2σ(*I*)
Radiation source: fine-focus sealed tube	*R*_int_ = 0.071
ω scans	θ_max_ = 24.8°, θ_min_ = 2.9°
Absorption correction: multi-scan (SADABS; Bruker, 2013)	*h* = −5→5
*T*_min_ = 0.785, *T*_max_ = 1.000	*k* = −13→13
16398 measured reflections	*l* = −31→33
2509 independent reflections	

### (II) 3-(2,4,5-Trimethyl-3,6-dioxocyclohexa-1,4-dienyl)propyl methacrylate Refinement

**Table:**

Refinement on *F*^2^	0 restraints
Least-squares matrix: full	Hydrogen site location: inferred from neighbouring sites
*R*[*F*^2^ > 2σ(*F*^2^)] = 0.049	H-atom parameters constrained
*wR*(*F*^2^) = 0.138	*w* = 1/[σ^2^(*F*_o_^2^) + (0.0609*P*)^2^ + 0.8066*P*] where *P* = (*F*_o_^2^ + 2*F*_c_^2^)/3
*S* = 1.04	(Δ/σ)_max_ < 0.001
2509 reflections	Δρ_max_ = 0.34 e Å^−^^3^
185 parameters	Δρ_min_ = −0.32 e Å^−^^3^

### (II) 3-(2,4,5-Trimethyl-3,6-dioxocyclohexa-1,4-dienyl)propyl methacrylate Special details

**Table:**

Geometry. All esds (except the esd in the dihedral angle between two l.s. planes) are estimated using the full covariance matrix. The cell esds are taken into account individually in the estimation of esds in distances, angles and torsion angles; correlations between esds in cell parameters are only used when they are defined by crystal symmetry. An approximate (isotropic) treatment of cell esds is used for estimating esds involving l.s. planes.

### (II) 3-(2,4,5-Trimethyl-3,6-dioxocyclohexa-1,4-dienyl)propyl methacrylate Fractional atomic coordinates and isotropic or equivalent isotropic displacement parameters (Å^2^)

**Table:**

	*x*	*y*	*z*	*U*_iso_*/*U*_eq_	
C1	0.1775 (5)	0.16524 (19)	0.14048 (8)	0.0209 (5)	
O1	0.0812 (4)	0.09197 (14)	0.11320 (6)	0.0324 (5)	
C2	0.3994 (5)	0.13528 (19)	0.18042 (8)	0.0200 (5)	
C21	0.4744 (6)	0.01196 (19)	0.18425 (8)	0.0277 (6)	
H21A	0.6438	0.0010	0.2079	0.042*	
H21B	0.5321	−0.0160	0.1534	0.042*	
H21C	0.2964	−0.0297	0.1939	0.042*	
C3	0.5109 (5)	0.21672 (19)	0.20975 (8)	0.0200 (5)	
C31	0.7283 (6)	0.1988 (2)	0.25224 (8)	0.0273 (6)	
H31A	0.6208	0.2090	0.2813	0.041*	
H31B	0.8945	0.2537	0.2516	0.041*	
H31C	0.8113	0.1221	0.2515	0.041*	
C4	0.4110 (5)	0.33563 (19)	0.20215 (8)	0.0211 (5)	
O4	0.5126 (4)	0.40980 (14)	0.22879 (6)	0.0306 (4)	
C5	0.1881 (5)	0.36569 (18)	0.16246 (8)	0.0194 (5)	
C51	0.1114 (6)	0.48890 (19)	0.15805 (9)	0.0280 (6)	
H51A	−0.0626	0.4987	0.1351	0.042*	
H51B	0.2866	0.5302	0.1471	0.042*	
H51C	0.0601	0.5182	0.1890	0.042*	
C6	0.0734 (5)	0.28445 (18)	0.13349 (8)	0.0189 (5)	
C7	−0.1420 (5)	0.3062 (2)	0.09083 (8)	0.0225 (5)	
H7A	−0.2806	0.3690	0.0979	0.027*	
H7B	−0.2666	0.2380	0.0839	0.027*	
C8	0.0333 (5)	0.33651 (19)	0.04742 (8)	0.0210 (5)	
H8A	0.1784	0.3979	0.0562	0.025*	
H8B	0.1526	0.2699	0.0384	0.025*	
C9	−0.1672 (5)	0.37361 (19)	0.00508 (8)	0.0208 (5)	
H9A	−0.3057	0.4343	0.0145	0.025*	
H9B	−0.0408	0.4035	−0.0199	0.025*	
O9	−0.3418 (3)	0.27759 (12)	−0.01293 (5)	0.0215 (4)	
C10	−0.5198 (5)	0.29825 (19)	−0.05223 (8)	0.0211 (5)	
O10	−0.5414 (4)	0.39082 (13)	−0.07045 (5)	0.0264 (4)	
C11	−0.6877 (5)	0.19585 (19)	−0.06980 (8)	0.0241 (6)	
C12	−0.6423 (7)	0.0922 (2)	−0.04544 (10)	0.0441 (8)	
H12A	−0.7486	0.0265	−0.0564	0.053*	
H12B	−0.5056	0.0883	−0.0182	0.053*	
C13	−0.8840 (6)	0.2102 (2)	−0.11027 (9)	0.0314 (6)	
H13A	−1.0002	0.1407	−0.1164	0.047*	
H13B	−0.7650	0.2273	−0.1376	0.047*	
H13C	−1.0239	0.2727	−0.1051	0.047*	

### (II) 3-(2,4,5-Trimethyl-3,6-dioxocyclohexa-1,4-dienyl)propyl methacrylate Atomic displacement parameters (Å^2^)

**Table:**

	*U*^11^	*U*^22^	*U*^33^	*U*^12^	*U*^13^	*U*^23^
C1	0.0196 (12)	0.0212 (12)	0.0220 (13)	−0.0030 (9)	0.0022 (10)	0.0003 (10)
O1	0.0395 (11)	0.0232 (9)	0.0328 (10)	−0.0028 (8)	−0.0109 (8)	−0.0067 (8)
C2	0.0204 (12)	0.0213 (12)	0.0183 (12)	−0.0005 (9)	0.0006 (10)	0.0029 (10)
C21	0.0338 (15)	0.0207 (13)	0.0281 (14)	0.0027 (10)	−0.0028 (11)	0.0001 (11)
C3	0.0186 (12)	0.0247 (12)	0.0167 (12)	−0.0011 (10)	0.0032 (9)	0.0030 (10)
C31	0.0297 (13)	0.0288 (14)	0.0230 (13)	−0.0012 (11)	−0.0026 (11)	0.0019 (11)
C4	0.0217 (12)	0.0229 (12)	0.0191 (12)	−0.0039 (10)	0.0031 (10)	−0.0021 (10)
O4	0.0364 (10)	0.0256 (9)	0.0286 (10)	−0.0042 (8)	−0.0065 (8)	−0.0071 (8)
C5	0.0194 (12)	0.0181 (12)	0.0208 (12)	0.0003 (9)	0.0028 (10)	0.0015 (10)
C51	0.0327 (15)	0.0214 (13)	0.0296 (15)	0.0021 (10)	0.0000 (11)	0.0013 (11)
C6	0.0162 (11)	0.0224 (12)	0.0183 (12)	0.0000 (9)	0.0016 (9)	0.0028 (10)
C7	0.0192 (12)	0.0252 (13)	0.0229 (13)	−0.0008 (10)	0.0000 (10)	0.0018 (10)
C8	0.0177 (12)	0.0229 (12)	0.0221 (13)	0.0005 (9)	−0.0016 (10)	−0.0006 (10)
C9	0.0205 (12)	0.0195 (12)	0.0221 (13)	−0.0023 (9)	−0.0027 (10)	0.0014 (10)
O9	0.0236 (9)	0.0190 (8)	0.0213 (9)	−0.0013 (6)	−0.0038 (7)	0.0000 (7)
C10	0.0212 (12)	0.0225 (13)	0.0194 (13)	0.0025 (10)	0.0009 (10)	−0.0023 (10)
O10	0.0314 (10)	0.0227 (9)	0.0244 (9)	−0.0005 (7)	−0.0049 (7)	0.0044 (7)
C11	0.0277 (13)	0.0227 (13)	0.0217 (13)	−0.0002 (10)	0.0006 (10)	−0.0018 (10)
C12	0.065 (2)	0.0248 (14)	0.0399 (17)	−0.0126 (13)	−0.0195 (14)	−0.0009 (13)
C13	0.0295 (14)	0.0303 (14)	0.0338 (15)	0.0014 (11)	−0.0036 (11)	−0.0079 (12)

### (II) 3-(2,4,5-Trimethyl-3,6-dioxocyclohexa-1,4-dienyl)propyl methacrylate Geometric parameters (Å, º)

**Table:**

C1—O1	1.220 (3)	C6—C7	1.510 (3)
C1—C2	1.491 (3)	C7—C8	1.531 (3)
C1—C6	1.494 (3)	C7—H7A	0.9900
C2—C3	1.345 (3)	C7—H7B	0.9900
C2—C21	1.500 (3)	C8—C9	1.509 (3)
C21—H21A	0.9800	C8—H8A	0.9900
C21—H21B	0.9800	C8—H8B	0.9900
C21—H21C	0.9800	C9—O9	1.448 (3)
C3—C4	1.487 (3)	C9—H9A	0.9900
C3—C31	1.505 (3)	C9—H9B	0.9900
C31—H31A	0.9800	O9—C10	1.342 (3)
C31—H31B	0.9800	C10—O10	1.213 (3)
C31—H31C	0.9800	C10—C11	1.490 (3)
C4—O4	1.224 (3)	C11—C13	1.401 (3)
C4—C5	1.489 (3)	C11—C12	1.416 (3)
C5—C6	1.342 (3)	C12—H12A	0.9500
C5—C51	1.501 (3)	C12—H12B	0.9500
C51—H51A	0.9800	C13—H13A	0.9800
C51—H51B	0.9800	C13—H13B	0.9800
C51—H51C	0.9800	C13—H13C	0.9800
			
O1—C1—C2	119.7 (2)	C1—C6—C7	116.11 (19)
O1—C1—C6	119.8 (2)	C6—C7—C8	110.83 (18)
C2—C1—C6	120.5 (2)	C6—C7—H7A	109.5
C3—C2—C1	119.6 (2)	C8—C7—H7A	109.5
C3—C2—C21	125.7 (2)	C6—C7—H7B	109.5
C1—C2—C21	114.71 (19)	C8—C7—H7B	109.5
C2—C21—H21A	109.5	H7A—C7—H7B	108.1
C2—C21—H21B	109.5	C9—C8—C7	113.78 (18)
H21A—C21—H21B	109.5	C9—C8—H8A	108.8
C2—C21—H21C	109.5	C7—C8—H8A	108.8
H21A—C21—H21C	109.5	C9—C8—H8B	108.8
H21B—C21—H21C	109.5	C7—C8—H8B	108.8
C2—C3—C4	119.7 (2)	H8A—C8—H8B	107.7
C2—C3—C31	125.6 (2)	O9—C9—C8	108.86 (17)
C4—C3—C31	114.7 (2)	O9—C9—H9A	109.9
C3—C31—H31A	109.5	C8—C9—H9A	109.9
C3—C31—H31B	109.5	O9—C9—H9B	109.9
H31A—C31—H31B	109.5	C8—C9—H9B	109.9
C3—C31—H31C	109.5	H9A—C9—H9B	108.3
H31A—C31—H31C	109.5	C10—O9—C9	114.78 (17)
H31B—C31—H31C	109.5	O10—C10—O9	122.9 (2)
O4—C4—C3	119.8 (2)	O10—C10—C11	124.7 (2)
O4—C4—C5	119.5 (2)	O9—C10—C11	112.39 (19)
C3—C4—C5	120.8 (2)	C13—C11—C12	124.3 (2)
C6—C5—C4	119.7 (2)	C13—C11—C10	116.3 (2)
C6—C5—C51	124.9 (2)	C12—C11—C10	119.4 (2)
C4—C5—C51	115.5 (2)	C11—C12—H12A	120.0
C5—C51—H51A	109.5	C11—C12—H12B	120.0
C5—C51—H51B	109.5	H12A—C12—H12B	120.0
H51A—C51—H51B	109.5	C11—C13—H13A	109.5
C5—C51—H51C	109.5	C11—C13—H13B	109.5
H51A—C51—H51C	109.5	H13A—C13—H13B	109.5
H51B—C51—H51C	109.5	C11—C13—H13C	109.5
C5—C6—C1	119.7 (2)	H13A—C13—H13C	109.5
C5—C6—C7	124.0 (2)	H13B—C13—H13C	109.5

### (II) 3-(2,4,5-Trimethyl-3,6-dioxocyclohexa-1,4-dienyl)propyl methacrylate Hydrogen-bond geometry (Å, º)

Cg is the centroid of the C1–C6 ring.

**Table:**

*D*—H···*A*	*D*—H	H···*A*	*D*···*A*	*D*—H···*A*
C9—H9*A*···O10^i^	0.99	2.72	3.624 (3)	153
C9—H9*B*···O10^ii^	0.99	2.70	3.595 (3)	150
C12—H12*A*···O1^iii^	0.95	2.53	3.422 (3)	156
C21—H21*A*···O4^iv^	0.98	2.52	3.455 (3)	161
C31—H31*C*···O4^iv^	0.98	2.68	3.638 (3)	166
C51—H51*B*···O10^v^	0.98	2.67	3.510 (3)	144
C31—H31*B*···*Cg*^ii^	0.98	2.95	3.534 (3)	119

Symmetry codes: (i) −*x*−1, −*y*+1, −*z*; (ii) *x*+1, *y*, *z*; (iii) −*x*−1, −*y*, −*z*; (iv) −*x*+3/2, *y*−1/2, −*z*+1/2; (v) −*x*, −*y*+1, −*z*.
